# Re-starting or initiating guideline-recommended hypoglycemic agents for patients admitted with hyperglycemic crisis

**DOI:** 10.3389/fmed.2025.1485357

**Published:** 2025-08-18

**Authors:** Abdulrahman I. Alshaya, Haya Al-Yahya, Abdulmajeed Alshehri, Mohammed Alrashed, Omar Alshaya, Lama Alfehaid, Hisham A. Badreldin

**Affiliations:** ^1^Department of Pharmaceutical Care Services, King Abdulaziz Medical City, Riyadh, Saudi Arabia; ^2^King Abdullah International Medical Research Center, Riyadh, Saudi Arabia; ^3^College of Pharmacy, King Saud bin Abdulaziz University for Health Sciences, Riyadh, Saudi Arabia

**Keywords:** DKA, HHS, diabetes, transition of care, hyperglycemic crises

## Abstract

**Introduction:**

Hyperglycemic crises, such as diabetic ketoacidosis (DKA) and hyperosmolar hyperglycemic state (HHS), are life-threatening complications of diabetes. This study aimed to assess the impact of early initiation of non-insulin hypoglycemic agents on glycemic variation following acute management of DKA/HHS.

**Research design and methods:**

This retrospective cohort study was conducted at King Abdulaziz Medical City and King Abdullah Specialized Children Hospital in Riyadh, Saudi Arabia. Patients with diabetes mellitus admitted between January 2015 and December 2023 were included if they had a confirmed diagnosis of DKA or HHS and received any non-insulin hypoglycemic agents after receiving acute care management. The primary outcome was to assess the impact of early initiation (defined as less than 24–48 h) of non-insulin hypoglycemic agents following acute management of DKA/HHS in controlling glycemic variation by measuring delta blood glucose “BG,” with secondary outcomes including hypoglycemia incidence, correctional insulin requirements, predictors for hospital length of stay (LOS), 90-day mortality, and hospital readmissions. Data was adjudicated by a separate clinician. Statistical analysis was performed using SPSS (IBM, Armonk, NY).

**Results:**

Out of 1,483 screened patients, 137 were included, experiencing a total of 226 hyperglycemic events. During hospitalization, 42.9% of patients were transitioned to oral hypoglycemic agents within 4 days. Transitioning to oral hypoglycemic medications resulted in a significant reduction in BG levels. Early re/initiation of hypoglycemic agents was strong predictor for shorter hospital LOS and lower 90-day mortality rate (2.1% vs. 10.1%, *p*-value = 0.02). There were no other significant outcomes.

**Conclusion:**

The study suggests that early initiation of non-insulin hypoglycemic results in similar delta BG compared to late initiation following acute management of DKA and HHS. The findings indicate that early transitioning to non-insulin hypoglycemic agents is associated with a lower 90-day mortality rate after acute management of DKA/HHS and a strong predictor for shorter hospital LOS. Further research, including randomized controlled trials, is recommended to validate these findings and explore long-term effects on mortality and clinical outcomes.

## Introduction

Hyperglycemic crises such as diabetic ketoacidosis (DKA) and hyperosmolar hyperglycemic state (HHS) are the most severe acute metabolic complications of diabetes that could be life-threatening if not treated promptly. Although the incidence of DKA is higher in type 1 (T1D) than in type 2 diabetic patients (T2D), T2D patients tend to present with more severe cases with higher mortality (1). The incidence of DKA is estimated to be 61.6 cases per 10,000 admissions in the United States (2). On the other hand, the prevalence of HHS is estimated to be near less than 1% of hospital admissions in diabetic patients with a ten times higher mortality rate than DKA cases (3).

According to a 2024 report by the International Diabetes Federation (IDF), Saudi Arabia ranks second in the region and seventh globally for diabetes prevalence. The report indicates that out of a population of 33.3 million, approximately 7 million individuals have diabetes, while an additional 3 million are classified as pre-diabetic. Saudi Arabia is one of the 21 countries and territories in the IDF MENA region, where 589 million people have diabetes worldwide, and this number is projected to rise to 163 million in the MENA region by 2050 (4). A study conducted in Saudi Arabia found that among adults aged 30 to 70 years, 23.7% have Diabetes Mellitus, and 14.1% have impaired fasting glucose, with incidence rates higher in urban areas (25.5%) compared to rural regions (19.5%) (5). If current trends continue, it is projected that by 2030, 50% of the population will be diabetic (6). Both type 1 and type 2 diabetes are prevalent in Saudi Arabia, with type 1 more common in children and adolescents and type 2 predominantly affecting adults, though there is significant overlap. This alarming incidence and prevalence of diabetes in Saudi Arabia contribute significantly to the increasing number of patients presenting to emergency departments with life-threatening complications, such as DKA and HHS (6).

Hyperglycemic Crises such occur due to insulin deficiency or resistance associated with increased levels of counterregulatory hormones such as glucagon and cortisol, leading to hyperglycemia, ketosis, dehydration, and electrolyte imbalance (7). Hence, the fundamental treatment of such hyperglycemic crises is fluid replacement, insulin therapy, correction of electrolyte imbalance, and treating underlying illness (8). Recently, multiple studies investigated the safety and efficacy of using different dosing strategies for insulin and fluids in DKA and HHS patients (9–13). DKA characterizes mainly by uncontrolled hyperglycemia [>13.9 mmol/L (>250 mg/dL)], metabolic acidosis, and presence of ketonemia and anion gap. Although HHS overlaps with DKA, it is associated with more severe hyperglycemia [>33.3 mmol/L (>600 mg/dL)] but no ketoacidosis.

Current guidelines suggest initiating institutional protocol to treat these emergency cases, yet it is still unclear when and how clinicians should re-start oral/injectable hypoglycemic agents in these patients. However, it is recommended that once DKA or HHS has resolved, patients should be transitioned to subcutaneous insulin or oral hypoglycemic agents in preparation for discharge. Structured discharge planning, thorough patient education, and detailed reconciliation of discharge medications are crucial for a safe transition of care and for preventing recurrence. Additionally, identifying factors that contributed to the hyperglycemic crisis, such as medication non-compliance and infections, is essential to prevent future episodes (12–15). Chronic diabetes management is quickly transforming as recent hypoglycemic agents such as sodium–glucose cotransporter 2 inhibitor (SGLT2i), glucagon-like peptide 1 receptor agonist (GLP-1 RA), showing mortality benefit compared to the old generation of hypoglycemic agents (12). Yet, the role of transition of care in these cases is yet to be explored in literature. Transition points, such as moving from critical care to acute care or a general floor, rehabilitation programs, and eventually returning to the community, present substantial challenges in medication therapy management (MTM). These transitions necessitate optimizing patients’ medication regimens and preventing adverse events associated with resuming home medications. Ensuring effective MTM during these phases is critical for maintaining therapeutic outcomes and minimizing risks (16, 17). This study aimed to assess the impact of early initiation of non-insulin hypoglycemic agents on glycemic variation following acute management of DKA/HHS.

## Methods

### Study design and setting

This was a retrospective cohort study of patients with diabetes mellitus who presented to King Abdulaziz Medical City (KAMC)—King Abdullah Medical City Specialist Hospital (KASCH) as part of the National Guard Hospital Affairs (NGHA) in Riyadh, Saudi Arabia. No formal treatment protocol was in place for the restarting or reinitiation of hypoglycemic agents, and all medication changes were made at the physician’s discretion. This study was approved from King Abdullah International Medical Center IRB with this reference number “NRC23R/014/01.” Due to the nature of the study as retrospective in nature, a waiver on the patient consent was implemented.

### Patient selection

Adult patients with a diagnosis of diabetes mellitus T2D identified via ICD 10 codes (E11.1. E12.1, E13.1, and E14.1) who were admitted to KAMC—KASCH between January 2015 and December 2023 were identified using electronic health record reporting of our institution. We conducted an independent examination of all patients with T2D admitted with DKA and HHS and eligible for oral/injectable hypoglycemic agents. Subjects were selected using random number generation until the desired sample size was reached or all available patients were included. Patients were excluded if they were diagnosed with type 1 diabetes, no documented DKA/HHS, ESRD on hemodialysis and those who could not be managed with oral/injectable hypoglycemic agents. This research received no specific grant from any funding agency in the public, commercial, or not-for-profit sectors. Patients or the public were not involved in the design, or conduct, or reporting, or dissemination plans of this research.

### Study variables and data collection

The primary outcome was to assess the impact of early initiation (defined as less than 24–48 h) of non-insulin hypoglycemic agents on glycemic variation following acute management of DKA/HHS. This was defined as the median delta blood glucose (BG) during the transition process (mg/dL). Delta blood glucose gap is calculated using the highest blood glucose value minus the lowest blood glucose value. The secondary outcomes were the median time to restart or initiate hypoglycemic agents, incidence of hypoglycemia (BG of 54–70 mg/dL), severe hypoglycemia (BG less than 54 mg/dL), requirement of corrective doses of insulin, infection during admission, hospital length of stay (LOS), Intensive Care Unit (ICU) LOS, in-hospital mortality, re-admission after 90 days, and 90 days all-cause mortality. Predictors of hospital readmission within 90 days for patients with hyperglycemic crises and predictors of prolonged hospital LOS in patients admitted with hyperglycemic crises were assessed using backward elimination method.

### Data analysis

**Figure 1:**
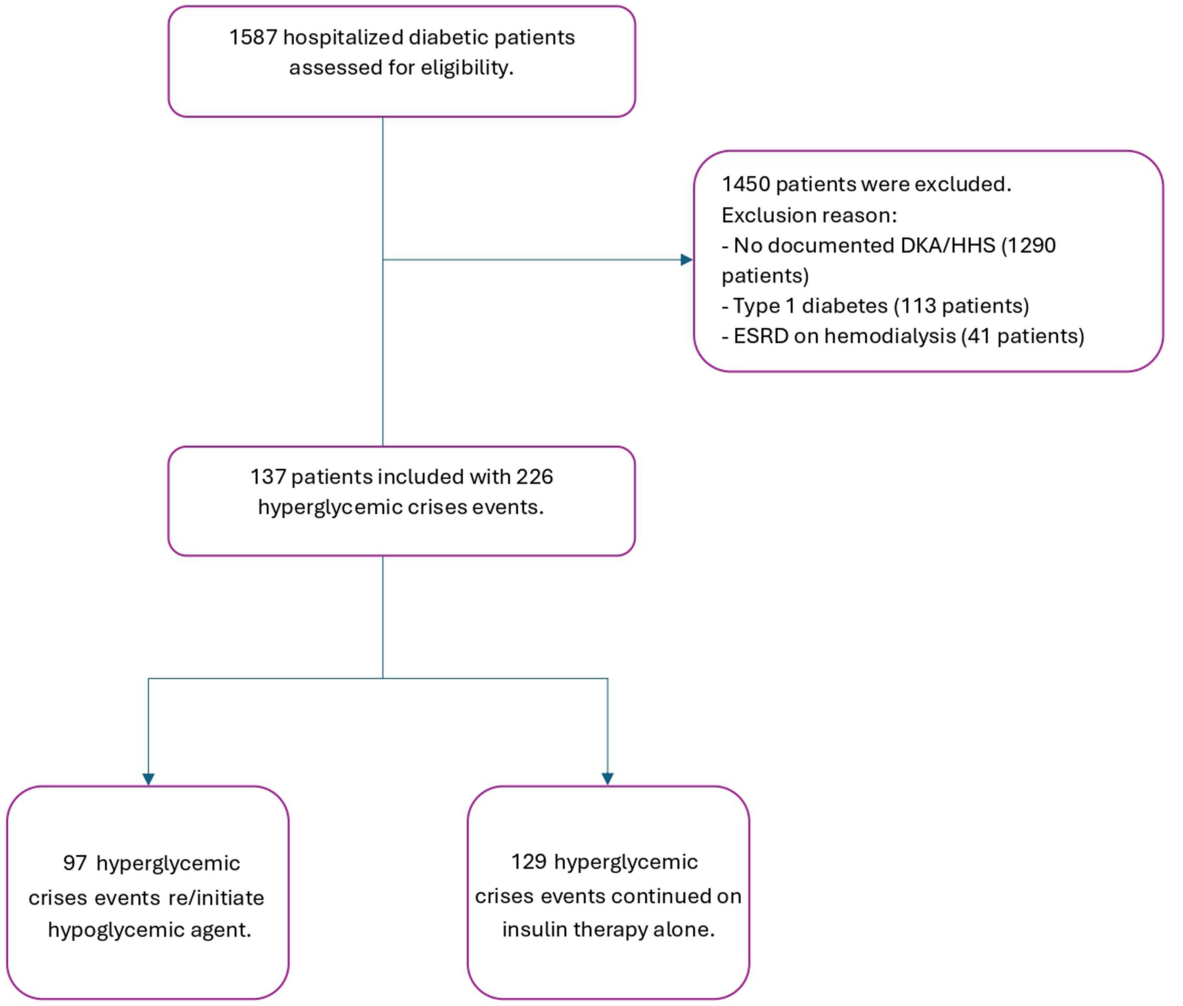
Study flowchart.

We hypothesized that delaying the initiation of guideline-recommended hypoglycemic agents would decrease the transition of care from critical to acute illness, thereby optimizing outcomes for diabetic patients. All patients who met the inclusion criteria were included in the study, and all data were collected from patients’ medical records (Figure 1). The collection sheet included (1) categorical variables, which were described as percentages, such as the presence of comorbidities and the type of hypoglycemic agents, and (2) numerical variables, which were calculated as mean ± standard deviation, such as baseline characteristics and laboratory values. All patients were followed and assessed for in-hospital mortality, re-admission after 90 days, and infections during admission. The duration of controlled blood glucose (BG) during hospitalization was determined by calculating the mean of daily BG measurements. The number of corrective insulin doses administered was recorded, including as needed insulin or any additional doses given outside of the patient’s standard insulin regimen.

The data were entered and saved into the Excel datasheet. For the study sample, a descriptive statistical analysis was conducted using variables that were summarized using means (SD), medians, and ranges. Continuous variables were presented as mean ± standard deviation or median interquartile range (IQR) as appropriate. Categorical variables were presented as number and percent. Student’s *t*-test or Mann–Whitney test were used to compare continuous variables as appropriate. Chi-square and Fisher exact test were used for categorical variables.

For the analysis of the major endpoint, a univariate analysis was done followed by a multivariable logistic regression model to adjust for any potential confounders. Additional variables selected *a priori* for inclusion in the model included age, gender, Atherosclerotic cardiovascular disease (ASCVD), heart failure, estimated glomerular filtration rate (eGFR) < 30 mL/min/1.73 m^2^, and BG level (mg/dL). A multivariable analysis was performed on risk factors that had a *p* < 0.1 on univariate analysis and variables known to affect the primary endpoint (24). An alpha error of 0.05 was considered significant for all analyses. All adjusted endpoints were reported as odds ratio with a 95% confidence interval for the major endpoint. Statistical significance defined by a *p*-value < 0.05 was reported for all minor endpoints. Statistical analysis was performed using SPSS (IBM, Armonk, NY).

## Results

### Participants characteristics

Among 1,483 patients with diabetes mellitus who were screened for DKA or HHS, 137 patients were included, with a total of 226 hyperglycemic events shown in the study flowchart (Figure 1). Of the included patients, males had more hyperglycemic crisis incidence than females, accounting for 58.41% of the study population. The median age was 68 years [interquartile range (IQR): 54–77]. Hypertension (75.22%) and chronic kidney disease (35.84%) were the most common comorbidities. The median hemoglobin A1c level was 10.9% (IQR: 8.8–13.2). The most frequently used home medication was insulin (75.11%), followed by metformin (36.2%) and dipeptidyl peptidase 4 inhibitors (DDPi) (18.14%) (Table 1).

**Table 1:** Demographic and clinical characteristics of patients admitted with hyperglycemic crisis.

Variable	Frequency/median (*n* = 226)	Percent/IQR
Female (*n*, %)	94	41.59%
Age (median, IQR)	68	54–77
BMI (kg/m^2^) (median, IQR)	26.2	22.2–31.1
Comorbidities		
HTN (*n*, %)	170	75.22%
ASCVD (n, %)	67	29.65%
CVA (*n*, %)	76	33.63%
HF (*n*, %)	36	15.93%
CKD (*n*, %)	81	35.84%
eGFR (mL/min/1.73m^2^) (median, IQR)	39	25–54
A1C (%) (median, IQR)	10.9	8.8–13.2
BG at admission (mg/dL)	525.6	417.6–702
Home medications		
Metformin (*n*, %)	82	36.2%
SGLT2i (*n*, %)	12	5.3%
GLP-1 (*n*, %)	4	1.8%
DDPi (*n*, %)	41	18.14%
Insulin (*n*, %)	166	75.11%
Sulfonylureas (*n*, %)	29	12.83%

IQR, interquartile range; BMI, body mass index; HTN, hypertension; ASCVD, atherosclerotic cardiovascular disease; CVA, cerebrovascular accident; HF, heart failure; CKD, chronic kidney disease; eGFR, estimated glomerular filtration rate; A1C, glycated hemoglobin; BG, blood glucose; SGLT2i, sodium-glucose co-transporter-2 inhibitors; GLP-1, glucagon-like peptide-1 receptor agonists; DDPi, dipeptidyl peptidase-4 inhibitors.

### Descriptive analysis and overall clinical outcomes

**Figure 2:**
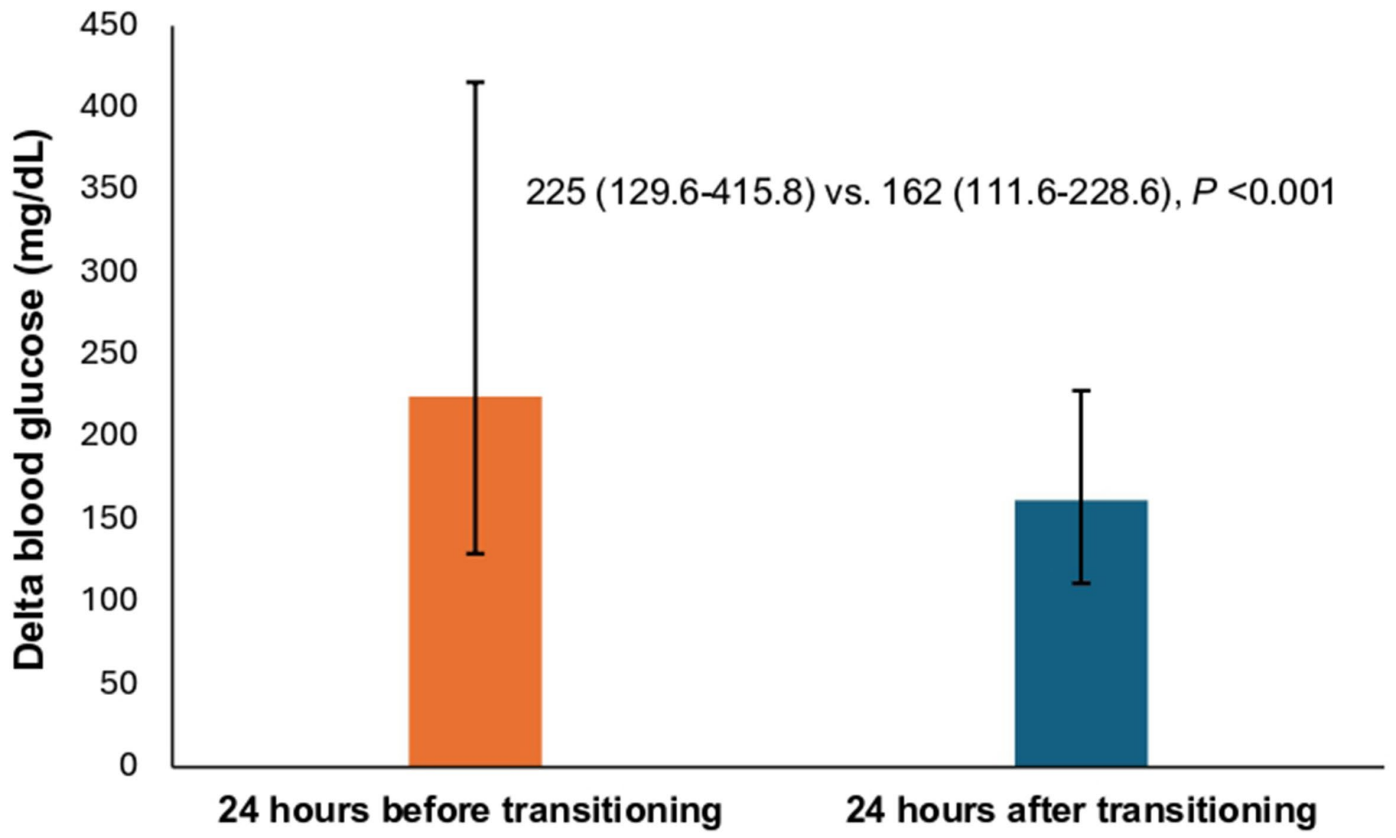
Comparison of the median delta blood glucose between patients during the transition process. * The height of each bar corresponds to the median value. Top error bar indicates quartile 3 (Q3). Bottom error bar indicates quartile 1 (Q1).

Primary outcome as defined by statistically significant decrease in delta blood glucose levels was observed during the transition process, with a median reduction of 63 mg/dL, from 225 mg/dL (24 h pre-transition) to 162 mg/dL (24 h post-transition) as shown in (Figure 2).

A total of 42.9% of admitted patients had been transitioned to oral agents other than insulin during hospitalization. Metformin (32.3%) and DDPi (20.8%) were the most commonly resumed/initiated hypoglycemic agent compared to other non-insulin hypoglycemic agents (Table 2). During hospitalization, the incidence of hypoglycemia (3.0–3.9 mmol/L) was 26.55%, with severe hypoglycemia (<3.0 mmol/L) occurring at a lower rate of 13.27%. Our analysis revealed that 42.73% of patients experienced readmission within 90 days. Moreover, 14.16% of the new DKA and HHS readmissions were attributed to acute complications of diabetes mellitus, DKA, or HHS. The cohort in this study was primarily treated on general medicine floors or spent less than 24 h in the ICU. During admission, patients experienced a median of 1 day (range: 0 to 2 days) of controlled blood glucose, with a median hospital length of stay of 5 days (range: 3 to 10 days). Additionally, 22 patients (9.73%) developed infections during their hospital stay. Additional data is also exhibited in (Figure 2; Table 2).

**Table 2:** Descriptive data and overall clinical outcome in patients admitted with hyperglycemic crisis.

Variable	Frequency/median (*n* = 226)	Percent/IQR
Patients were transitioned to oral/injectable hypoglycemic medications (*n*, %)	97	42.92%
Time to re/initiate hypoglycemic agents (days) (median, IQR)	4	2–6
In-hospital metformin (*n*, %)	73	32.3%
In-hospital SGLT2i (*n*, %)	9	3.98%
In-hospital GLP-1 (*n*, %)	0	0.00%
In-hospital DDPi (*n*, %)	47	20.80%
In-hospital TZD (*n*, %)	1	0.44%
In-hospital SU (*n*, %)	14	6.19%
Days of controlled blood glucose during admission (median, IQR)	1	0–2
Hospital LOS	5	3–10
Hypoglycemia (3.0–3.9 mmol/L) (*n*, %)	60	26.55%
Severe hypoglycemia (<3.0 mmol/L) (*n*, %)	30	13.27%
Complication (infection during admission) (*n*, %)	22	9.73%
Hospital mortality (*n*, %)	3	1.33%
90-days mortality after discharge (*n*, %)	15	6.73%
Re-admission within 90 days (for any reason) (*n*, %)	94	42.73%
Readmission due to DKA/HHS/DM complication (*n*, %)	32	14.16%

IQR, interquartile range; LOS, length of stay; SGLT2i, sodium-glucose co-transporter-2 inhibitors; GLP-1, glucagon-like peptide-1 receptor agonists; DDPi, dipeptidyl peptidase-4 inhibitors; TZD, thiazolidinediones; SU, sulfonylureas; DKA, diabetic ketoacidosis; HHS, hyperosmolar hyperglycemic state; DM, diabetes mellitus.

When comparing outcomes based on the use of hypoglycemic agents during hospitalization, patients receiving oral hypoglycemic medications had a lower 90-day post-discharge mortality rate compared to those on scheduled insulin 2.1 and 10.1%, respectively (*p* value = 0.02). Notably, no differences in hypoglycemia and severe hypoglycemia were noted between the two groups (Table 3).

**Table 3:** Comparison of outcomes based on the use of oral hypoglycemic agents during hospital admission.

Outcomes	Use of oral hypoglycemic agent during admission (*n* = 97)	Use of scheduled insulin during admission (*n* = 129)	*p*-value
Hypoglycemia (3.0–3.9 mmol/L) (*n*, %)	29 (29.9%)	31 (24%)	0.323
Severe hypoglycemia (<3.0 mmol/L) (*n*, %)	13 (13.4%)	17 (13.17%)	0.961
90-days mortality after discharge (*n*, %)	2 (2.1%)	13 (10.1%)	0.015
Hospital mortality (*n*, %)	0 (0%)	3 (2.3%)	0.162
Re-admission within 90 days (for any reason) (*n*, %)	36 (37.1%)	58 (44.96%)	0.236
Number corrective doses of insulin (median, IQR)	3 (2–7)	4 (1–6)	0.907
Hospital length of stay (days) (median, IQR)	5 (3–11)	5 (4–8)	0.803
Days of controlled blood glucose during admission (median, IQR)	1 (0–2)	1 (0–2)	0.613

**Figure 3:**
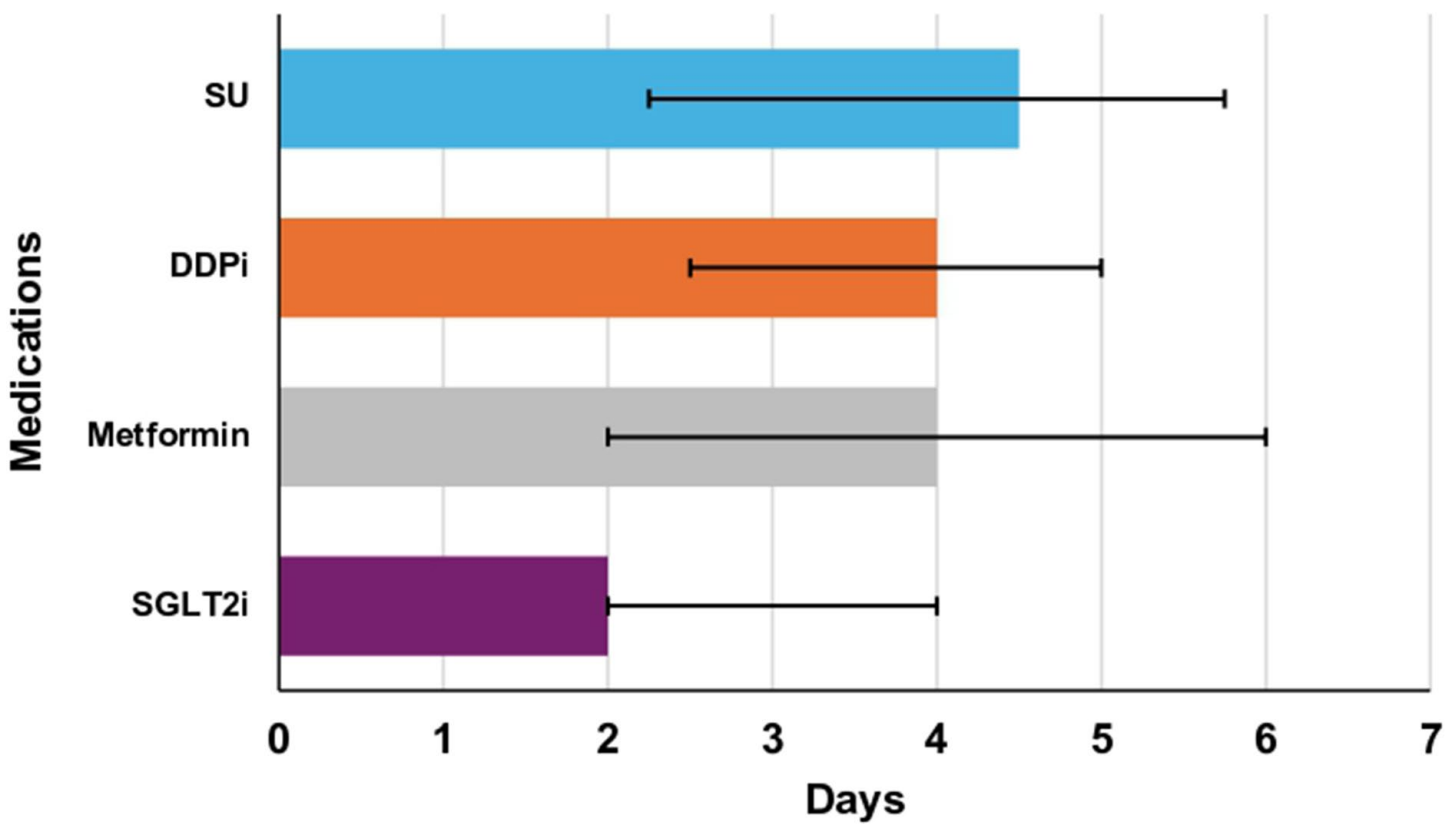
Median time to re/initiate oral agents in days (Q1-Q3). Metformin: 4 days (2–6), sodium-glucose cotransporter-2 inhibitors (SGLT2i): 2 days (2–4), dipeptidyl peptidase inhibitors (DDPi): 4 days (2.5–5), sulfonylureas (SU): 2.25 days (4.5–5.75). There was one patient restarted on thiazolidinediones and is not illustrated in this figure. * The length of each bar corresponds to the median value. The right error bar indicates quartile 3 (Q3), and the left error bar indicates quartile 1 (Q1).

In this study, analysis of the data, as shown in (Table 4), revealed that a longer delay in initiating or re-initiating oral hypoglycemic medications (Figure 3), along with the occurrence of both hypoglycemia and severe hypoglycemia, were identified as independent predictors of a prolonged length of hospital stay. Additionally, age emerged as a predictor of hospital readmission within 90 days for patients with hyperglycemic crises [odds ratio (OR): 1.029; 95% confidence interval (CI) 1.007–1.050; *p*-value 0.008] (Table 5).

**Table 4:** Predictors of prolonged hospital LOS in patients admitted with hyperglycemic crises.

Variable	Coefficient	95% confidence interval	*p*-value
Age (years)	0.024	−0.145–0.194	0.776
A1c %	0.211	−0.863–1.284	0.697
Blood glucose at admission (mg/dL)	−0.017	−0.035–0.002	0.074
eGFR <30 (mL/min/1.73 m^2^)	−1.476	−9.524–6.572	0.716
Time to re/initiate hypoglycemic agents (days)	1.148	0.901–1.395	0.000
Hypoglycemia (54–70 mg/dL)	8.226	2.077–14.375	0.009
Severe hypoglycemia (<54 mg/dL)	10.274	1.992–18.555	0.016

A1c, glycated hemoglobin; eGFR, estimated glomerular filtration rate.

**Table 5:** Predictors of hospital readmission within 90 days for patients with hyperglycemic crises.

Variable	Odds ratio	95% confidence interval	*p*-value
Age	1.029	1.007–1.050	0.008
Female gender	0.924	0.511–1.669	0.792
ASCVD	1.178	0.593–2.341	0.640
Heart failure	1.567	0.701–3.500	0.274
eGFR < 30	1.686	0.896–3.174	0.105
Transitioned to oral hypoglycemic agent before discharge	0.819	0.450–1.489	0.513

ASCVD, atherosclerotic cardiovascular disease; eGFR, estimated glomerular filtration rate.

## Discussion

This study was designed to identify the outcomes resulting from the transition of care in diabetic patients admitted with DKA/HHS to guideline-directed medical therapy. Antidiabetic medications can be used alone or alongside low-dose basal insulin, potentially offering a safer and more effective approach compared to traditional basal-bolus insulin regimens. This study further supports the existing evidence that less severe hyperglycemic crises can be managed outside of the intensive care unit (ICU). This approach can substantially reduce therapy costs and reserve ICU beds for more critical cases (18–20). Although nearly 50% of patients initiated treatment with OHAs, it is crucial to note that this does not indicate that OHA was the only treatment regimen. Many patients may have continued using insulin alongside OHAs, suggesting a combined therapy approach rather than a complete transition. This distinction is essential for accurately interpreting our results and understanding post-DKA/HHS treatment strategies.

The most important finding is the association of a delay in restarting or initiating oral antidiabetic medications, coupled with the occurrence of hypoglycemia, as significant predictors of a prolonged hospital stay. This emphasizes the importance of timely medication initiation and effective glycemic control to optimize patient outcomes. Multiple studies have indicated that intensive insulin therapy is associated with a higher risk of hypoglycemia (21, 22). This increased risk has been linked to greater morbidity and mortality among hospitalized patients (23, 24). Consequently, while insulin therapy remains a recommended strategy for managing hyperglycemia in hospitalized patients, concerns about hypoglycemia have prompted leading professional organizations worldwide to revise their glucose target recommendations and antidiabetic medication selections (25, 26).

Consistent with previous research found that 100% of hospitalized type 1 diabetic patients using insulin experienced hypoglycemic episodes, and 99.4% of type 2 diabetic patients using insulin had at least one episode of hypoglycemia (27). The DIAMOND study indicated that hypoglycemia events were more frequent in patients on sliding scale insulin therapy, suggested a strict monitoring of blood glucose levels (28). Additionally, a pilot randomized controlled study that compared sitagliptin monotherapy and its combination with basal insulin in hospitalized type 2 diabetic patients, demonstrating comparable efficacy and safety profiles for both treatment strategies (29). Another larger non-inferiority trial confirmed these results when compared sitagliptin plus basal insulin to a basal-bolus insulin regimen, with no significant differences observed in hospital stay duration or hypoglycemia rates (30).

Together these findings provide direct support for existing guidelines that recommend strict monitoring of blood glucose levels to prevent hypoglycemia in diabetic patients. The American Diabetes Association (ADA) and the American Association of Clinical Endocrinologists (AACE) recommend monitoring blood glucose levels every hour in critically ill patients receiving intravenous insulin infusion (25). There were no significant differences in the incidence of hypoglycemia between oral hypoglycemic agents and scheduled insulin administration during hospitalization, which may be attributed to frequent blood glucose monitoring in hospital settings. Additionally, our institution’s protocol mandates a blood glucose check before insulin administration, which might have lowered the rate of hypoglycemia reported with insulin use. It is important to note that these findings are specific to the inpatient setting and may not extrapolate to outpatients who continue insulin therapy after discharge. This approach may be particularly beneficial for older patients, who are at higher risk for hospital acquired infections.

Our findings suggest a potential benefit of using oral hypoglycemic agents during hospitalization. Notably, a statistically significant difference emerged in 90-day post-discharge mortality rates between patients who transitioned to oral hypoglycemic agents and those on scheduled insulin therapy with no differences in hypoglycemia and severe hypoglycemia. The observed lower mortality rate might be attributable to the superior safety profile of oral medications relative to insulin. Additionally, the convenient administration of oral medications may have enhanced patient adherence post-discharge, and mitigated the common problem of non-compliance that may contributed to their initial hospitalization. Although insulin is recognized for its rapid and effective glycemic control, there is a general patient preference for avoiding injectable treatments.

Our analysis of participants’ characteristics indicated that older patients experiencing hyperglycemic crises are more likely to be readmitted to the hospital within 90 days. This highlights the importance of developing age-specific management approaches for this vulnerable group. The transition to oral hypoglycemic medications in the management of hyperglycemic crises may be a suitable treatment option for hospitalized geriatric patients, providing comparable outcomes to scheduled insulin administration. A randomized controlled study conducted in long-term care facilities reported no statistical difference in the rate of in-hospital complications, emergency room visits, or mortality when comparing oral hypoglycemic medications to insulin. Interestingly, while fasting blood glucose levels were comparable between groups, patients on insulin therapy exhibited higher mean daily blood glucose levels relative to those treated with oral hypoglycemic medications (31).

It is noteworthy that the use of GLP-1 RAs and SGLT-2 is as home medications in this study was minimal (1.8% vs. 5.3%). Even after the resolution of hyperglycemic crises, their usage remained low (0.00% vs. 3.98%), despite recent guideline recommendations endorsing these medications as first-line therapies due to their cardiorenal benefits and glucose-lowering effects (31, 32). This low utilization is consistent with other reports in the literature highlighting the need to increase their use (33, 34). Moreover, our current evaluation revealed a prevalence of hyperglycemic crises of 9.6% of the screened patients with type 2 DM (137 out of 1,427 patients). This prevalence is higher than the United States average annual rate of DKA of 6.3% from 2000 to 2014 in all types of DM (34, 35).

### Strengths, limitations, and future research directions

A significant strength of our study lies in its novel approach to investigating the initiation or resumption of guideline-directed oral medical therapy following hyperglycemic crises among hospitalized type 2 diabetic patients. This research provides new insights into the comparative effectiveness of oral hypoglycemic medications versus scheduled insulin therapy in this specific context. Our findings contribute to the existing body of knowledge by demonstrating the potential benefits of oral hypoglycemic medications in lowering mean daily blood glucose levels without increasing the risk of hypoglycemia. Unlike previous studies, which have primarily focused on insulin therapy for managing hyperglycemia in hospitalized patients, our study highlights the efficacy and safety of oral hypoglycemic agents as an alternative treatment strategy. This unique perspective allows for a more comprehensive understanding of hyperglycemic crisis management and suggests potential new avenues for treatment. However, the study has limitations due to its relatively small sample size, which may impact the generalizability of our findings. Further research with larger cohorts is warranted to validate these results. Additionally, the retrospective design of our study may have resulted in missing data, potentially introducing bias. Future randomized controlled studies are recommended to investigate the long-term effects of restarting or initiating oral hypoglycemic agents compared to scheduled insulin on mortality and other patient-oriented clinical outcomes. Moreover, research is necessary to identify optimal strategies for preventing medication delays and reducing the risk of hypoglycemia after resolving hyperglycemic crises. Another limitation of this study is that patients with end-stage renal disease on hemodialysis (ESRD on HD) were excluded, potentially limiting generalizability to more severe presentations of hyperglycemia crises. Additionally, we observed a low utilization of newer therapies such as glucagon-like peptide-1 receptor agonists (GLP-1 RAs) and sodium-glucose cotransporter-2 inhibitors (SGLT-2i) which limited our ability to assess the impact of these medications on the outcomes.

## Conclusion

The study suggests that early initiation of non-insulin hypoglycemic results in similar delta BG compared to late initiation following acute management of DKA and HHS. The findings indicate that early transitioning to non-insulin hypoglycemic agents is associated with a lower 90-day mortality rate after acute management of DKA/HHS and a strong predictor for shorter hospital LOS. Additionally, transitioning to oral hypoglycemic medications resulted in a significant reduction in BG levels. The study suggests that early initiation of oral hypoglycemic agents could be a viable alternative to insulin in transitional care for hyperglycemic crises. Further research, including randomized controlled trials, is recommended to validate these findings and explore long-term effects on mortality and clinical outcomes.

## Data Availability

The raw data supporting the conclusions of this article will be made available by the authors, without undue reservation.

## Glossary

ADA: American Diabetes Association
AACE: American Association of Clinical Endocrinologists
ASCVD: Atherosclerotic cardiovascular disease
BG: Blood glucose
CKD: Chronic kidney disease
CVA: Cerebrovascular accident
DPPi: Dipeptidyl peptidase-4 inhibitors
DKA: Diabetic ketoacidosis
eGFR: Estimated glomerular filtration rate
HHS: Hyperosmolar hyperglycemic state
HF: Heart failure
IQR: Interquartile range
LOS: Length of stay
OHA: Oral hypoglycemic agents
PLOS: Prolonged length of stay
SGLT2i: Sodium-glucose co-transporter-2 inhibitors
GLP-1: Glucagon-like peptide-1 receptor agonists
T2D: Type 2 diabetes
HE: Hyperglycemic emergency
MTM: Medication therapy management
OR: Odds ratio
CI: Confidence interval
